# The Pseudoalveolar Form of Sarcoidosis: A Diagnostic Trap Not to Be Ignored

**DOI:** 10.7759/cureus.32375

**Published:** 2022-12-10

**Authors:** Chaynez Rachid, Saad Bounhar, Lamyae Amro

**Affiliations:** 1 Service de Pneumologie, Hôpital Arrazi, Centre Hospitalier Universitaire Mohammed VI, Marrakech, MAR

**Keywords:** systemic granulomatosis, corticosteroids, chronic condensations, non-necrotizing granuloma, pulmonary sarcoidosis

## Abstract

Sarcoidosis is a benign systemic granulomatosis of unknown etiology. Interstitial parenchymal involvement is typical. The pseudoalveolar form is atypical, often acute in onset, and diﬃcult to diagnose; however, it rapidly improves with corticosteroid therapy. Here, we report a case of pseudoalveolar sarcoidosis with distinct and confusing radiological and clinical presentation in a young female patient. Through this work, we emphasize the rarity of this pseudoalveolar form, the diﬃculty of making an early diagnosis, and the importance of considering it early. Finally, we discuss the excellent response of this unusual form of sarcoidosis to corticosteroid therapy and the importance of starting therapy early.

## Introduction

Sarcoidosis is a systemic granulomatous disease with an unknown etiology, with the most common form being mediastino-pulmonary sarcoidosis. The most common manifestations of the disease are the involvement of the hilar and mediastinal lymph nodes. The pseudoalveolar form is uncommon, frequently acute, and may be isolated or associated with mediastinal lymph node involvement. This form is easily confused with various respiratory diseases, resulting in frequent diagnostic delays. Histology forms the base and the rest of the factors support the diagnosis. Although spontaneous regression is possible, the disease can be chronic and can be effectively treated by oral corticosteroid therapy.

## Case presentation

**Figure 1:**
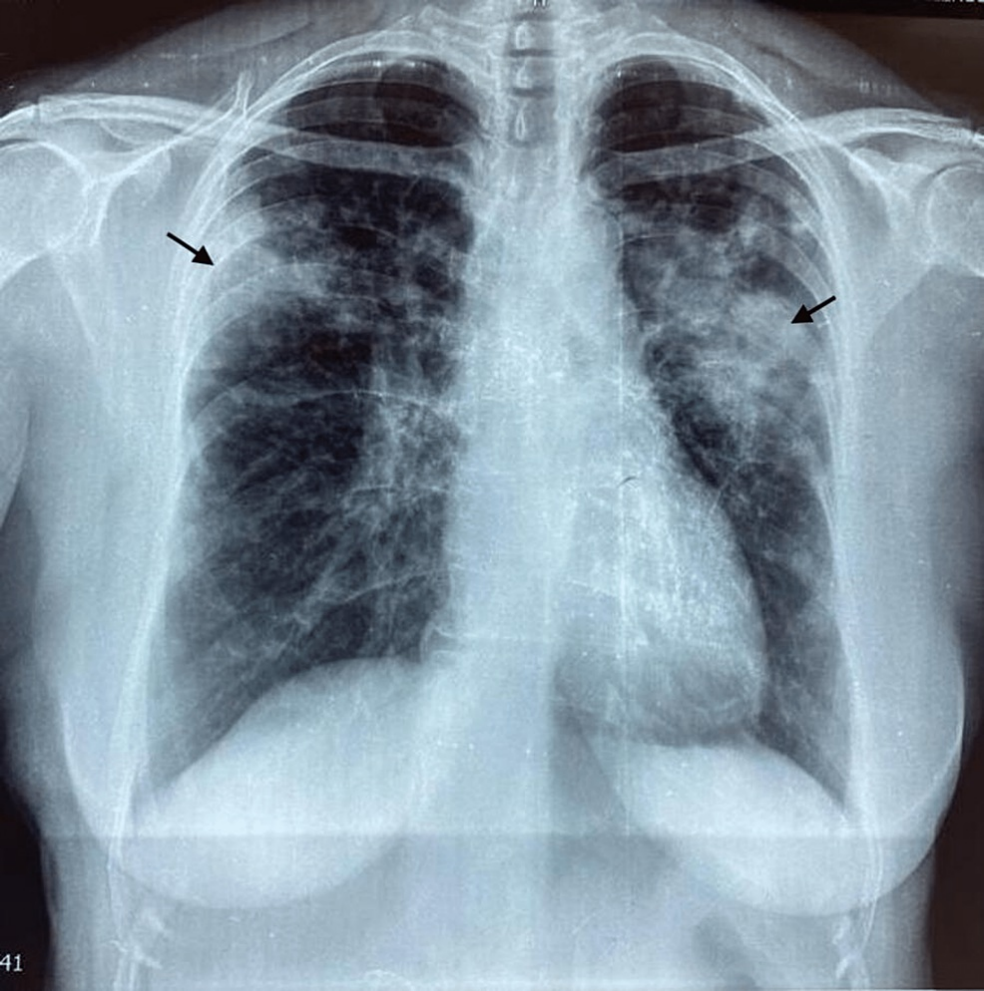
Posteroanterior chest radiograph demonstrates bilateral alveolar syndrome with foci condensation. The image shows homogeneous dense opacity, poorly limited left upper, a dense opacity located in the subpleural level of the right upper lobe extended bilaterally, and opacities of nodular type in the right.

Here, we present the case of a 50-year-old Black female with no significant history and no professional or environmental exposure. Six months before her admission, she presented with a dry cough, dyspnea on exertion, and polyarthralgia of inﬂammatory appearance in the context of feverish sensations but conservation of the general state. Clinical examination of the pleura and lungs showed a bilateral condensation syndrome associated with increased vocal vibrations, maturity, and crackling rales. The chest X-ray showed an alveolar syndrome of homogeneous dense opacity poorly limited in the perihilar left, dense opacity in the subpleural level of the right upper lobe, and right nodular opacities (Figure 1).

**Figure 2:**
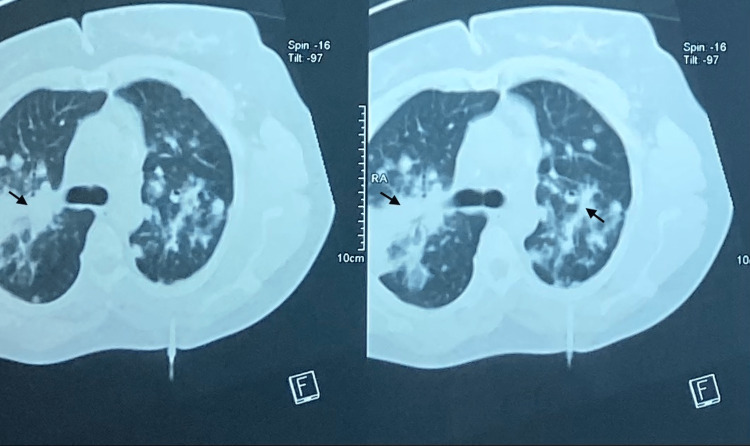
Chest computed tomography scan in the parenchymal window showing foci of alveolar consolidation. The images show confluent parenchymatous condensations in bilateral hilar patches with a pseudomass appearance and diffuse nodular lesions in both the pulmonary and subpleural fields.

Sputum microscopy and sputum Xpert gene were negative. The sedimentation rate was 88 mm/hour in the first hour. The hemogram showed hyperleukocytosis of 12,000/mm^3^ and lymphopenia of 14,150/mm^3^. After 10 days of antibiotic therapy (amoxicillin + clavulanic acid: 3 g/day), no clinical improvement or radiographic changes were noted. The thoracic computed tomography (CT) scan showed bilateral hilar opacities with a pseudomass appearance with diffuse nodular lesions in both the pulmonary and subpleural fields and bilateral mediastinal-hilar adenopathies with calcifications (Figure 2).

**Figure 3:**
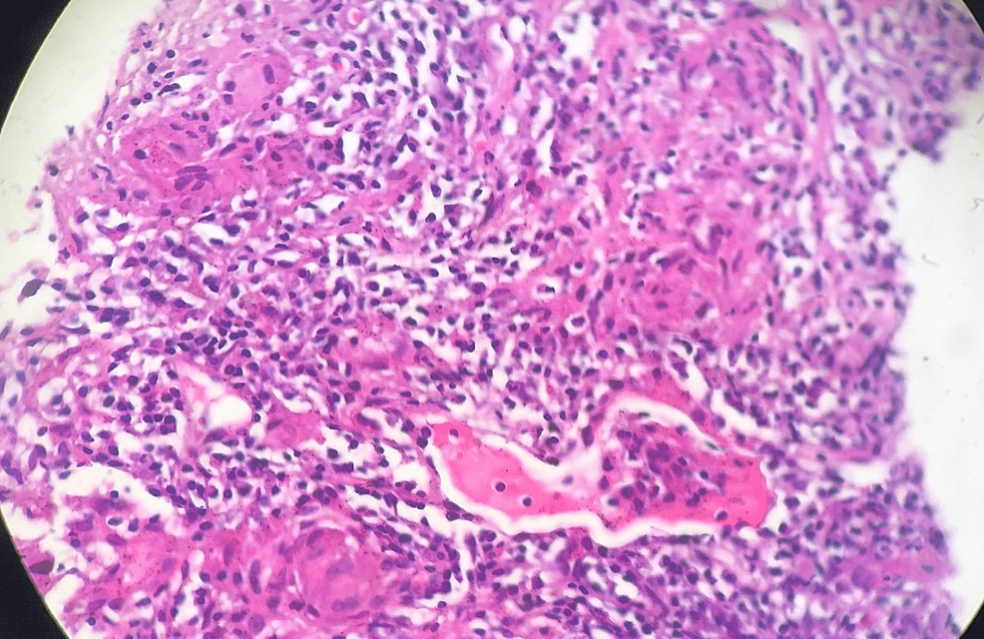
Tuberculoid granulomatous inflammatory lesion. Microscopic examination shows a dense thickened fibrous tissue hyalinized in places and infiltrated by mono and polynucleated inflammatory elements with epithelial and giant cells forming nodules without true necrosis and histological signs of malignancy (hematoxylin and eosin, 4×).

Bronchoscopy showed thick spurs in the right upper lobar bronchus with no visible buds and granulomas, but the left bronchi were unremarkable. Staged bronchial biopsies showed bronchial mucosa reworked by a non-specific chronic inflammatory lesion in a discrete acute flare, with no definite diagnosis or malignancy. Cytology was hematic and discreetly inflammatory, without suspicious cells. The search for an acid-alcohol-resistant bacillus in the bronchial aspirates was negative, and the bronchoalveolar lavage (BAL) showed a lymphocytic predominance. Transbronchial biopsy showed granulomatous inflammation consisting of mono and polynucleated cells and epithelioid giant cells without caseation (Figure 3).

The angiotensin-converting enzyme assay showed a serum activity of 62 IU/L. The phosphocalcic balance showed normal blood calcium and phosphorus levels with a high urinary phosphorus level of 1,454.00 mg/L or 1,366.76 mg/24 hours. The biopsy of the salivary glands showed a discrete and non-specific interstitial sialadenitis of degree 1 of Chisholm and Mason. Corticosteroid therapy with prednisone 40 mg/day was started with regression of dyspnea and respiratory function abnormalities after one month, with the radiographic abnormalities beginning to clear. Spirometry was performed as part of the follow-up assessment, showing a flow-volume curve at the limit of normal with a forced vital capacity of 81% and a Tiffeneau ratio of 97% (Table 1).

**Table 1:** Spirometry follow-up assessment. The table shows a flow-volume curve at the limit of normal with an FVC of 81% and a Tiffeneau ratio of 97%. FVC = forced vital capacity; FEV1 = forced expiratory volume in the first second

FVC	FEV1	FEV1/FVC	DEP
81%	92%	97%	103%

## Discussion

Sarcoidosis is an inflammatory, multisystemic disease of unknown cause with a wide range of clinical manifestations. Although the disorder can affect virtually any organ in the body, it predominantly affects the lungs, lymphatic system, skin, eyes, or a combination of these sites. It is characterized by the formation of non-caseating granulomas [1]. Atypical manifestations, such as the pseudoalveolar form, can indicate a range of differential diagnoses, causing a diagnostic delay.

In our study, we report the pseudoalveolar form of sarcoidosis in a 50-year-old Black woman. This is in agreement with the literature, which indicates a female predominance and a peak at 45-65 years, corresponding to the perimenopausal peak incidence [2].

Pseudoalveolar sarcoidosis may be revealed by a non-regressive pneumonia presentation, despite adequate antibiotic therapy [3]. This offers a set of diagnostic hypotheses, notably mycobacterial infections, especially in a region with high prevalence. Many paraclinical investigations are necessary before the diagnosis of sarcoidosis is posed [4]. In our case, in view of the alveolar opacity in chest X-rays, inflammatory syndrome, and lymphopenia, antibiotics were administered for 10 days, with no clinical or radiological improvement. Because of a lack of response, sputum bacilloscopy and molecular mycobacterial testing were negative. Later, a search of mycobacterial infections during a bronchoscopy was negative.

The CT findings in pulmonary sarcoidosis are very broad, with the most common lesions being diffuse micronodules with irregular contours associated with a lymphatic distribution. A CT scan can reveal alveolar condensation or ground glass. In rare cases where these images are in the foreground, conjunction with bilateral lymphadenopathy and micronodules are highly suggestive of the diagnosis. CT also helps to distinguish reversible inflammatory lung lesions under treatment (nodules, confluent nodules, and alveolar condensation) from irreversible fibrous lesions (retraction and honeycomb) [5].

In our case, the CT scan showed confluent parenchymatous condensations in bilateral hilar patches with a pseudomass appearance with diffuse nodular lesions in both pulmonary and subpleural fields and bilateral mediastinal-hilar adenopathies with calcifications, thus pointing to a pseudoalveolar form of sarcoïdosis.

The most typical histological finding is the tuberculous non-caseous granuloma [4,6]. Positive histological findings are found in 80-90% of sarcoidosis cases.

The CT-guided biopsy showed the presence of a tuberculoid granulomatous inflammatory lesion composed of mono and polynucleated cells with epithelioid and giant cells forming nodules without caseous necrosis.

Bronchial fibroscopy represents a critical examination, allowing bronchial and transbronchial biopsies. The BAL preferentially oriented toward the territories affected makes it possible to eliminate other etiologies of the chronic alveolar syndrome (eosinophil lung) and shows a comparable cell profile to the usual forms of sarcoidosis. In our case, the BAL showed a lymphocytic predominance [7,8].

All biological, histological, and radiological findings, eliminating other causes of granulomatous etiologies, led to the diagnosis of sarcoidosis. The excellent evolution under corticosteroids during follow-up further confirmed the diagnosis.

## Conclusions

Pulmonary sarcoidosis in its pseudoalveolar form is rare and rather confusing, constituting a diagnostic trap that should not be ignored in respiratory pathology. We emphasize the rarity of this form and the difficulty of establishing the diagnosis, along with the importance of thinking about it early, especially when there is a discrepancy between the extent of the alveolar syndrome and the noisy nature of the clinical symptoms. We also propose a review of the literature concerning this atypical form of sarcoidosis.

## References

[1] Drent M, Crouser ED, Grunewald J (2021). Challenges of sarcoidosis and its management. N Engl J Med.

[2] Gerke AK, Judson MA, Cozier YC, Culver DA, Koth LL (2017). Disease burden and variability in sarcoidosis. Ann Am Thorac Soc.

[3] Bulbul F, Koca I, Tamam L, Demirkol ME, Cakmak S, Ersahinoglu E (2020). The prevalence of sarcopenia in bipolar disorder. Neuropsychiatr Dis Treat.

[4] (1999). Statement on sarcoidosis. Joint Statement of the American Thoracic Society (ATS), the European Respiratory Society (ERS) and the World Association of Sarcoidosis and Other Granulomatous Disorders (WASOG) adopted by the ATS Board of Directors and by the ERS Executive Committee, February 1999. Am J Respir Crit Care Med.

[5] Criado E, Sánchez M, Ramírez J, Arguis P, de Caralt TM, Perea RJ, Xaubet A (2010). Pulmonary sarcoidosis: typical and atypical manifestations at high-resolution CT with pathologic correlation. Radiographics.

[6] Valeyre D, Freynet O, Naccache JM, Carton Z, Nunes BH (2011). Sarcoidose grave. EMC Pneumol.

[7] Pedro C, Melo N, Novais E Bastos H (2019). Role of bronchoscopic techniques in the diagnosis of thoracic sarcoidosis. J Clin Med.

[8] Gharsalli H, Mlika M, Sahnoun I, Maalej S, Douik El Gharbi L, Mezni FE (2018). The utility of bronchoalveolar lavage in the evaluation of interstitial lung diseases: a clinicopathological perspective. Semin Diagn Pathol.

